# The clinical implications and interpretability of computational medical imaging (radiomics) in brain tumors

**DOI:** 10.1186/s13244-025-01950-6

**Published:** 2025-03-30

**Authors:** Yixin Wang, Zongtao Hu, Hongzhi Wang

**Affiliations:** 1 Department of Brain Oncology, Hefei Cancer Hospital, Chinese Academy of Sciences, Hefei, P. R. China; 2 Anhui Province Key Laboratory of Medical Physics and Technology, Institute of Health and Medical Technology, Hefei Institutes of Physical Science, Chinese Academy of Sciences, Hefei, P. R. China

**Keywords:** Radiomics, Brain tumor, Interpretability, Deep learning, Artificial intelligence

## Abstract

**Abstract:**

Radiomics has widespread applications in the field of brain tumor research. However, radiomic analyses often function as a ‘black box’ due to their use of complex algorithms, which hinders the translation of brain tumor radiomics into clinical applications. In this review, we will elaborate extensively on the application of radiomics in brain tumors. Additionally, we will address the interpretability of handcrafted-feature radiomics and deep learning-based radiomics by integrating biological domain knowledge of brain tumors with interpretability methods. Furthermore, we will discuss the current challenges and prospects concerning the interpretability of brain tumor radiomics. Enhancing the interpretability of radiomics may make it more understandable for physicians, ultimately facilitating its translation into clinical practice.

**Critical relevance statement:**

The interpretability of brain tumor radiomics empowers neuro-oncologists to make well-informed decisions from radiomic models.

**Key Points:**

- Radiomics makes a significant impact on the management of brain tumors in several key clinical areas.
- Transparent models, habitat analysis, and feature attribute explanations can enhance the interpretability of traditional handcrafted-feature radiomics in brain tumors.
- Various interpretability methods have been applied to explain deep learning-based models; however, there is a lack of biological mechanisms underlying these models.

**Graphical Abstract:**

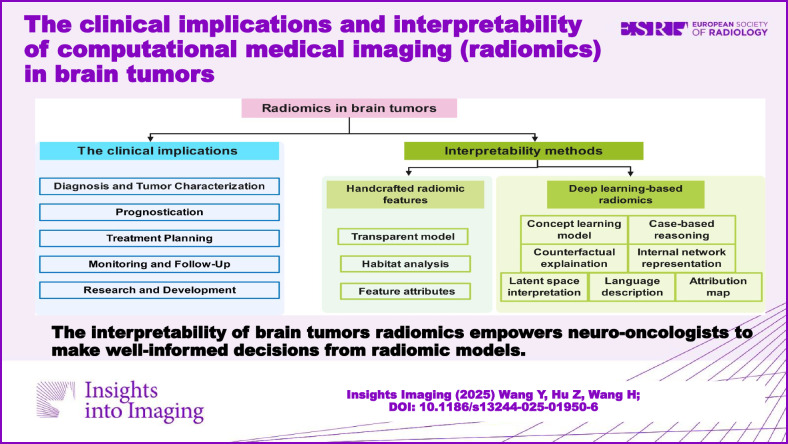

## Introduction

Brain tumors are abnormal cell growths in the brain or central spinal canal. They are classified into primary brain tumors, which originate in the brain [1], and metastatic brain tumors, which spread from other parts of the body [2]. Conventional imaging techniques like magnetic resonance imaging (MRI) and computed tomography scans are crucial for diagnosing, characterizing, and planning treatment for brain tumors [3]. However, these techniques lack the ability to provide detailed quantitative information, limiting clinicians’ ability to fully assess tumor characteristics.

Radiomics involves extracting numerous features from medical images using advanced computational methods [4]. It allows for quantitative assessment of tissue characteristics and provides noninvasive biomarkers, making it widely used in brain tumor research [5]. Radiomics enhances tumor characterization, improves diagnostic and prognostic accuracy, personalizes treatment, and advances our understanding of tumor biology through noninvasive imaging.

Radiomics can be categorized into two types: traditional radiomics, which uses image processing and statistical methods to extract features, and deep learning (DL) radiomics which employs DL algorithms like convolutional neural network (CNN) to automatically extract high-level features [6]. While radiomics has shown good performance in brain tumor studies, the complex algorithms can act as a “black box” [6], making it difficult for physicians to understand the process, thereby impeding its translation into clinical applications.

Interpretability bridges the gap between humans and decision-making systems, providing explanations that are both accurate and comprehensible. Many explainable artificial intelligence(AI) techniques have been used in brain tumor radiomic studies [7], but most overlook the importance of the ‘target audience.’ Interpretability means explaining models in terms understandable to humans. Domain experts, such as medical doctors, need to trust the radiomic models and gain medical insights from them.

We will review recent clinical applications of radiomics in brain tumors. We will also discuss the interpretability of computational medical imaging in brain tumors and share our views on future research needed for better acceptance of radiomics.

## The clinical implications of radiomics in brain tumor

**Fig. 1:**
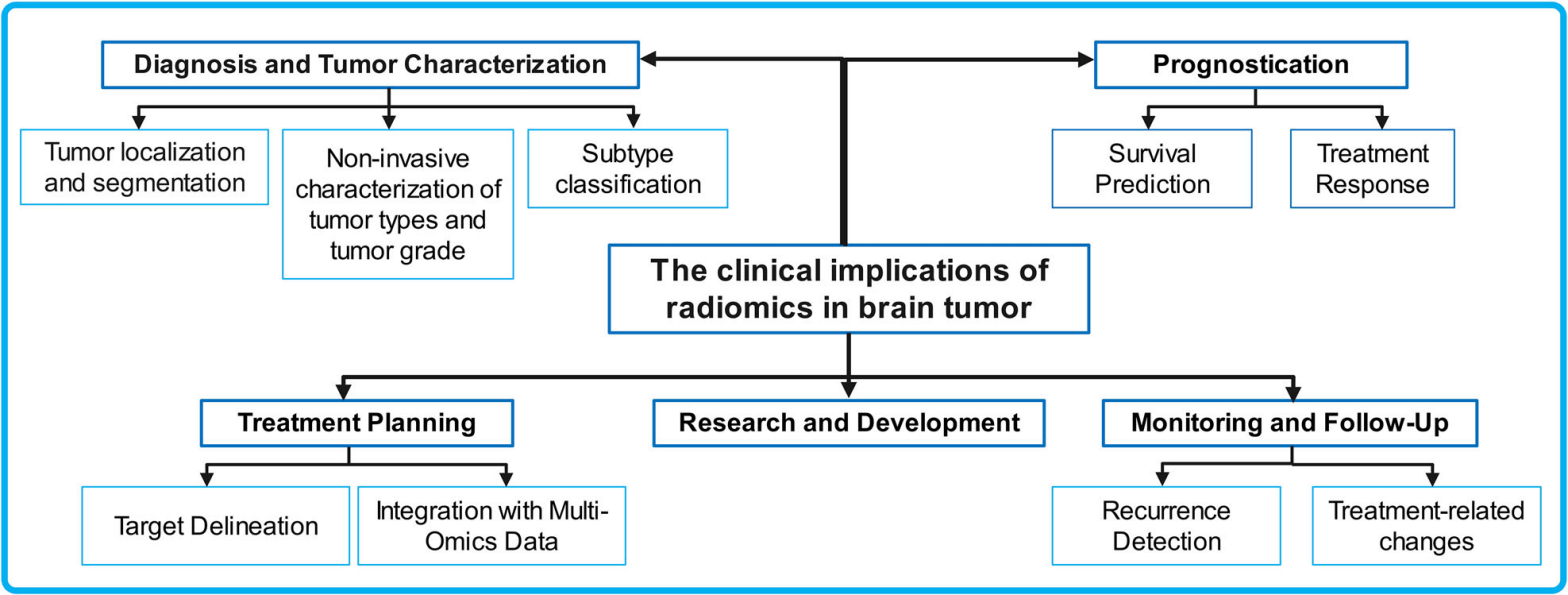
The clinical applications of radiomics in brain tumor

Radiomics extracts numerous quantitative features from medical images using data-characterization algorithms, offering valuable clinical insights. In brain tumors, radiomics significantly impacts key areas of management, including diagnosis, treatment planning, and prognosis. The clinical applications of radiomics in brain tumor is shown in Fig. 1.

## Diagnosis and tumor characterization

### Tumor localization and segmentation

Radiomics could identify precise brain tumor location, followed by segmentation to delineate tumor boundaries from healthy tissue. CNN and other DL models proved effective for tumor segmentation. Xue et al developed a DL model using a 3D U-Net with adaptations in training, testing strategies, network structures, and model parameters for brain tumor segmentation, achieving high prediction accuracy for both low-grade glioma and glioblastoma (GBM) patients [8] Sebastian et al developed a single multi-task CNN to predict genetic features and automatically segment the T2w-hyperintense region, achieving a mean whole tumor Dice score of 0.84 [9]. Automated radiomic segmentation ensured consistent results and enhanced tumor visualization.

### Noninvasive characterization of tumor types and tumor grade

Although the gold standard for diagnosing brain tumors involves histopathological analysis, stereotactic biopsies, or resection, these techniques are invasive and limited by spatial sampling variability. Radiomics offered a noninvasive way to distinguish between different types of brain tumors, such as gliomas and brain metastasis (BM) [10–12]. Dongming et al analyzed tumor habitat characteristics in gliomas, primary central nervous system lymphoma, and BM, achieving an area under the curve (AUC) of 0.91 and an accuracy of 76.9% in the test cohort [13]. Whole-slide images (WSIs) also proved useful in classifying adult-type diffuse gliomas. Wang et al developed an integrated diagnosis model for the classification of gliomas from annotation-free WSIs, achieving an AUC of 0.90 [14]. Additionally, radiomics accurately distinguished between low-grade and high-grade tumors [9, 15–17], critical for formulating personalized treatment strategies tailored to tumor aggressiveness.

### Subtype classification

Advanced radiomic techniques identified molecular signatures or tumor subtypes, such as IDH mutation status in gliomas [18–20]. Houneida et al used MRI to develop and assess a model for detecting O6-methylguanineDNA methyltransferase (MGMT) promoter methylation in GBM via transfer learning [21]. Jing et al used pretreatment 18F-DOPA positron emission tomography-based radiomics to predict MGMT status, achieving 80% accuracy [22]. Hongbo et al evaluated DL in multiparametric MRI-based radiomics, with the DL radiomic signature demonstrating superior discriminative power for predicting telomerase reverse transcriptase promoter mutations, yielding AUCs of 0.890 in external validation cohorts [23]. Ye Li et al developed a radiomic model to predict epidermal growth factor receptor (EGFR) resistance mutations in BM [24], while Yanran Li et al created a T1-CE signature to identify EGFR mutations and human EGFR2 status in brain adenocarcinoma, assisting in treatment planning [25]. Radiomics integrated with molecular biology for brain tumor molecular subtypes classification, advancing precision medicine in neuro-oncology.

## Prognostication

### Survival prediction

By leveraging advanced imaging analytics, radiomics could measure tumor heterogeneity, correlating with biological aggressiveness and treatment response. This approach could predict overall survival (OS) and progression-free survival (PFS) in brain tumor patients, aiding in risk stratification. Asma et al developed a radiomics model to classify glioma patients into short-term, medium-term, or long-term survivors [26]. Anna et al evaluated radiomics and DL-based models on the LUNG1 dataset to predict 2-year OS in non-small cell lung cancer (NSCLC), achieving AUCs of 0.67, 0.63, and 0.67 for radiomic, deep, and combined features, respectively, and 0.64 for direct CNN classification [27].

### Treatment response

Radiomics provides detailed insights into treatment effectiveness, guiding more personalized and effective therapeutic strategies. Ying et al developed radiomics signatures from MRI of BM scans to predict the response to EGFR-tyrosine kinase inhibitors in NSCLC patients with BM, achieving an AUC of 0.808 in an external validation cohort [28]. Jingwei et al preoperatively predicted MGMT methylation status based on MRI radiomics and validated its value for evaluating the effect of temozolomide chemotherapy. The results showed that the radiomics approach successfully divided patients into high-risk and low-risk groups for OS after temozolomide chemotherapy [29]. Yixin et al developed a pretreatment MRI-based radiomic-clinical model to assess the treatment response of whole-brain radiotherapy, yielding AUCs of 0.851 for identifying responders and non-responders in the validation cohort [30]. For stereotactic radiosurgery (SRS) in BM patients, studies showed that radiomics could prospectively identify patients who were insensitive to SRS therapy [31, 32].

## Treatment planning

### Target delineation

Radiomic features highlight subtle tissue differences, improving target delineation in radiotherapy to optimize tumor dose while sparing healthy tissue [33, 34]. Prasanna et al developed a radiomics-based CNN (RadCNN) using multimodal MRI to segment both high- and low-grade gliomas into different subcompartments [35]. Ying et al developed an automatic segmentation method using holistically nested neural networks, achieving a Dice similarity coefficient of 0.78 and sensitivity of 0.81 in the Brain Tumor Image Segmentation (BRATS) 2013 high-grade glioma dataset [36].

### Integration with multi-omics data

Radiomics, integrated with clinical, genetic, and molecular data, offers a comprehensive approach to understanding brain tumor biology. Reum et al analyzed immune cell numbers per unit volume of core tumor tissue in high-grade gliomas to correlate immune microenvironment characteristics with clinical prognosis and radiomic signatures, showing good performance in predicting immune phenotypes [37]. Zizhuo et al identified prognostically relevant immune features and used MRI radiomics to predict the OS of lower-grade glioma patients and their response to immune checkpoints, potentially guiding precision medicine for immunotherapy [38]. Guanzhang et al developed a preoperative MRI-based radiomics model to predict glioma survival and linked key radiomic features to biological functions using RNA-seq, single-cell sequencing, and immunohistochemical staining, revealing associations with immune response [39].

## Monitoring and follow-up

Radiomics offers detailed, noninvasive assessments for brain tumor monitoring, enabling treatment adjustments based on tumor response and potentially improving outcomes.

### Recurrence detection

Jun et al used radiomic features from diffusion-weighted imaging (DWI) and arterial spin labeling to differentiate between radiation-induced brain injury (RIBI) and tumor recurrence in glioma patients [40]. Leihao et al constructed a clinic-radiomics model combining age, extent of resection, Ki-67 index, surgical history, and radiomics signature for recurrence prediction in atypical meningiomas, achieving an AUC of 0.840 [41]. Zijian et al developed a radiomic model using delta radiomics and a RUSBoost classifier to distinguish radiation necrosis from tumor progression in BM after Gamma Knife radiosurgery, achieving 73.2% predictive accuracy [42].

### Treatment-related changes

Radiomics could distinguish between tumor recurrence and treatment-related changes, such as radiation necrosis and white matter alterations [43]. Ismail et al used quantitative 3D shape features of enhancing lesions to capture pathophysiologic differences between pseudoprogression and tumor recurrence. The results showed that differential expression patterns might be due to white matter structure alterations via infiltration, resulting in surface shape irregularities [44].

With advances in radiotherapy technology, the accuracy and conformability of radiotherapy significantly improved. However, brain white matter changes were more sensitive to radiation [45], leading to RIBI, affecting patients’ quality of life [46]. Mingming et al used multisequence MR radiomics to analyze brain white matter subtle changes before and after radiotherapy, linking radiomic feature changes to radiation doses, with significant features found in the 5–10 Gy, 20–30 Gy, and 30–40 Gy dose gradients [47], potentially guiding dose escalation for radiation oncologists.

## Research and development

Radiomics helps stratify patients for clinical trials, optimize trial design, and provide quantitative endpoints, but the lack of standardization and focus on interpretability has limited their use in brain tumor trials. Franco et al used the prospective SPORT trial to investigate a 1H MR-spectroscopy sequence within a radiomics analytics pipeline. By combining deep autoencoder and linear discriminant models, they developed a classification algorithm capable of predicting the origin of a brain lesion and the IDH genotype from magnetic resonance spectroscopy, achieving an overall accuracy of over 90% [48]. Another clinical trial evaluated radiomics for brain tumor characterization. Hollon et al developed a label-free optical imaging method and deep CNN to predict diagnosis in near real-time during surgery. They demonstrated that CNN-based diagnosis of stimulated raman histology images was non-inferior to the pathologist-based interpretation of conventional histologic images, with overall accuracies of 94.6% and 93.9%, respectively [49].

## The interpretability of radiomics in brain tumor

With the increasing use of AI algorithms in brain tumor radiomics, the amount and complexity of available tumor information have risen steadily. However, most studies did not aim to interpret the mechanisms of their models [50].

**Fig. 2:**
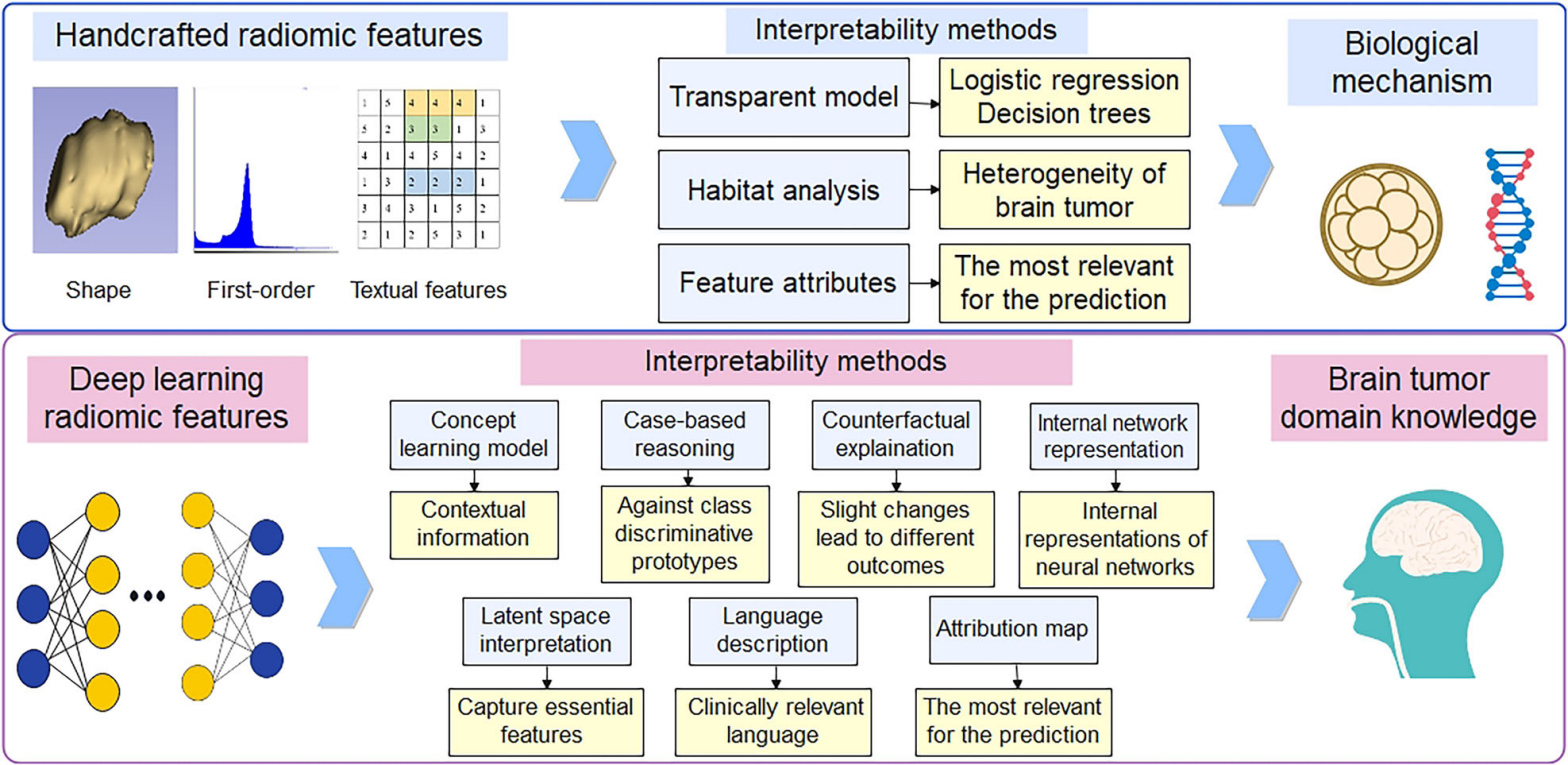
The main steps and interpretability methods of traditional radiomics and DL radiomics

Traditional radiomics use handcrafted features extracted from lesion segmentation on images. The main steps include data acquisition and preprocessing, tumor segmentation, feature extraction and selection, and using machine learning (ML) methods for modeling [6, 51]. DL radiomics aims to directly learn feature extraction from the entire raw image using an end-to-end approach [6, 51]. DL radiomics often being considered “black boxes” due to the complexity of their learned feature extractors [52–54]. In contrast, traditional radiomics are more interpretable, as the features are explicitly defined and extracted based on predetermined formulas [55]. The main steps and interpretability methods of traditional radiomics and DL radiomics are shown in Fig. 2.

**Fig. 3:**
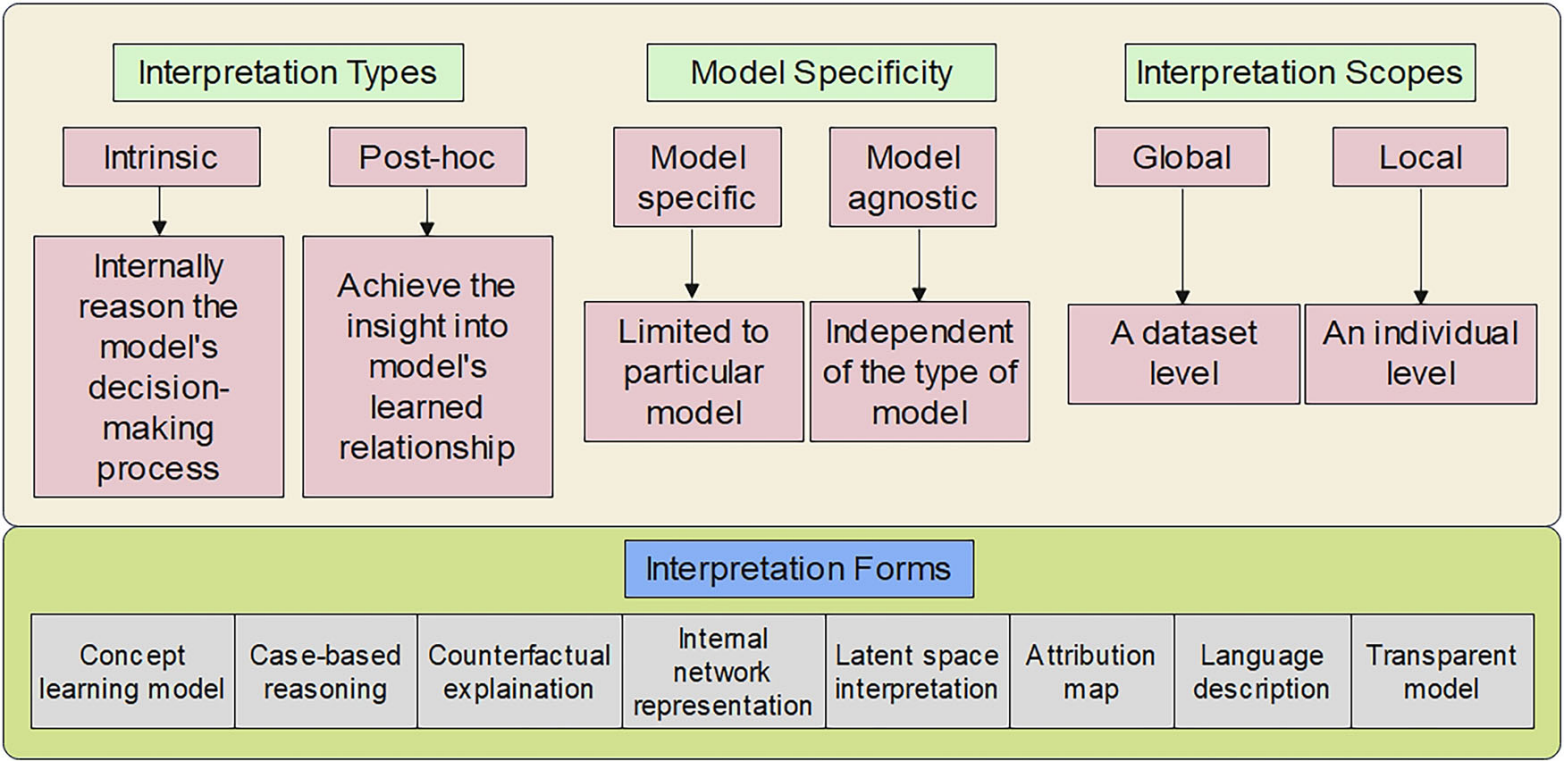
The criteria and the corresponding categories of explainable artificial intelligence

Interpretable AI may address this interpretability challenge [7, 52–54, 56]. The criteria and the corresponding categories are shown in Fig. 3.

Transparent models are structured to be understandable, such as linear regression models or decision trees model (with more details in Table 1) [57–60]. The advantage of transparent models is that they provide intrinsic interpretability; the model structure itself explains how the outputs are generated from the inputs. Post hoc explanations are obtained by external methods. These explanations are not part of the model itself but are generated externally. These methods are used when dealing with complex models that are not inherently interpretable. Model-specific approaches rely on particular structures of the model and include all transparent models or gradient-based methods for neural networks. The advantage of model-specific approaches is their ability to provide detailed and tailored explanations, but they lack generality, as they cannot be applied to other types of models. Model-agnostic approaches have no special requirements for the model. The flexibility of model-agnostic methods makes them versatile for different models, but they may require additional computation and sometimes lack the precision of model-specific explanations. Local explanations focus on variables that contribute to a specific decision, these explanations focus on understanding the contribution of each input feature to the outcome for that particular case. Conversely, global explanations explain the interaction mechanisms of the model variables, and the main benefit of global explanations is that global explanations provide a broad understanding of the model’s decision-making process, which is useful for validating the model and ensuring compliance with regulations. After reviewing the literature, we identify various interpretability methods for traditional and DL radiomics of brain tumors, based on the type of generated explanations and technical similarities. Considering that the “target audience” of this review might be clinical oncologists, we also emphasize interpretability based on domain knowledge.

**Table 1:** The application of transparent models in radiomics

Transparent models	Logistic regression	Decision trees
How it works	Logistic regression estimates the likelihood of an outcome, such as tumor malignancy, by using weighted input features like shape and intensity. These weights, known as coefficients, are easily interpretable, as they reflect the extent to which each feature influences the predicted result.	A decision tree divides data according to specific feature thresholds (e.g., size > 2 cm), forming a structure similar to a flowchart. Each node corresponds to a decision based on a particular feature, and tracing the branches leads to a final prediction.
Example for clinicians	When a feature such as “tumor texture” has a positive coefficient, it indicates that an increase in texture value corresponds to a higher probability of malignancy. This clarity enables clinicians to grasp the model’s predictions intuitively.	If the initial split in a decision tree is based on “ brain tumor size > 2 cm,” followed by a second decision on “shape irregularity > 0.8,” the model provides a clear explanation of how it reached a malignancy conclusion. This level of clarity is consistent with the decision-making processes used in clinical practice.

## The interpretability of traditional radiomics in brain tumor

### Habitat analysis

**Fig. 4:**
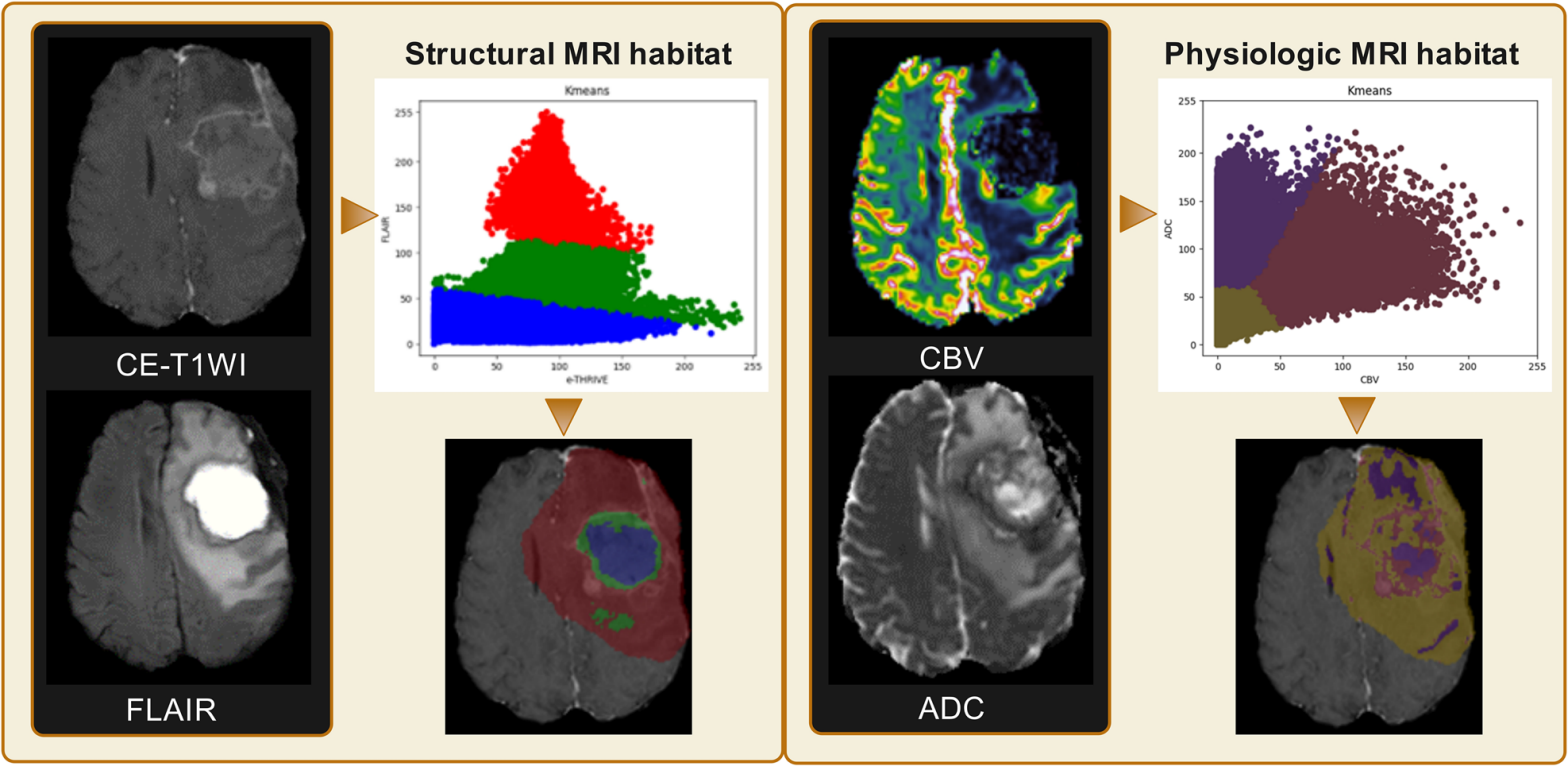
Habitat analysis differentiates subregions within a heterogeneous tumor by identifying similar voxels with the same tumor biology

Habitats are tumor regions characterized by distinct imaging features from intrinsic cell populations or local environmental conditions. Multiple types of imaging biomarkers facilitate habitat analysis [61], as shown in Fig. 4, and make the extracted radiomic features more biologically meaningful. Habitat analysis does not explain the ML models themselves; therefore, we provide biological context in this section.

Verma et al used multimodal MRI to delineate habitat regions, including the necrotic core, enhancing lesion, and extracted radiomic features to predict PFS in GBM patients [62]. However, they did not elaborate on the pathological mechanisms linking these features to PFS prognosis. Ruchika et al identified radiomic features from tumor habitats that were prognostic for PFS and assessed their morphologic basis with corresponding histopathologic attributes in GBM [63]. They found that Laws features from enhancing tumors were associated with infiltrating tumors and hyperplastic blood vessels, CoLIAGe inverse different moment features correlated with the perinecrotic zone, and wave-wave-edge Laws features were linked to the outermost section of the resected tumor [63]. Niha et al developed a radiomic risk score (RRS) using 25 Gd-T1w MRI features to predict PFS in GBM. They also linked RRS features to molecular pathways involved in cell differentiation, adhesion, angiogenesis, and chemoresistance in GBM [64].

Based on clinicians’ experience, brain tumors are divided into regions rich in pathophysiological information, giving radiomic features deeper significance. However, current modeling approaches are too simplistic and do not fully utilize the advantages of habitat analysis. More interpretive methods could be combined for further exploration.

### Explaining using feature attributes

The three main types of feature attribution methods (with more details in Table 2) are activation-based, gradient-based, and perturbation-based [7]. Activation-based and gradient-based methods are commonly utilized in DL models; therefore, this section focuses on perturbation-based methods.

**Table 2:** How feature attribution methods operate in radiomics and deliver valuable insights to clinicians

Feature attribution methods	Activation-based (e.g., Grad-CAM)	Gradient-based (e.g., Saliency Maps)	Perturbation-based (e.g., LIME)
How it works	Highlights the regions of the image most important to the prediction by analyzing activations in specific neural network layers.	Computes how changes in the input affect the output by analyzing the gradients of the prediction with respect to input features.	Perturbs the input data and observes how the model’s prediction changes to identify important features.
Valuable insights to clinicians	Grad-CAM might reveal that the model focused heavily on the tumor’s irregular margins or core region, providing the clinician with insight into the model’s reasoning. This alignment with clinical expectations helps clinicians trust the AI’s decision.	Saliency map could highlight the tumor’s specific areas with unusual density, demonstrating that these regions were critical for the malignancy prediction. This helps verify whether the model is focusing on the appropriate anatomical features typically associated with malignancy, like irregular borders or heterogeneous texture.	LIME could indicate that a slight smoothing of the “tumor texture” feature would have reduced the predicted likelihood of recurrence. This interpretation offers valuable insights to the clinician about the aggressiveness of the tumor.

Perturbation-based methods reveal how models made predictions by probing them with different inputs and analyzing the input-output pairs. These methods are post hoc and model-agnostic. Local interpretable model-agnostic explanations (LIME) generate new data points similar to the instance being explained, observe the model’s behavior on these perturbed instances, and then fit a simple model to highlight important features [65, 66]. Xiuying et al provided an automated glioma grading platform based on ML models and used LIME to reveal important parameters for grading. They found that, besides Ki-67, morphological features like the standard deviation of cells’ max axis and perimeter significantly contributed to glioma grading, as high-grade glioma often exhibited strong heterogeneity with irregular cell shapes [67]. Georgios et al used MRI-based radiomics to predict IDH mutations in gliomas, employing LIME and Shapley additive explanations (SHAP) to reveal that radiomic features were associated with voxel size variability, texture disorder, gray level spread, and contrast in gray values [68]. These interpretations provided understandable patterns between IDH mutation status and the extracted features.

SHAP is a unified framework of six methods used for interpreting predictions, defining a class of additive feature importance measures and theoretical results [65]. Wang et al utilized SHAP to validate a radiomic-clinical model assessing the treatment response of whole-brain radiotherapy [30]. The SHAP summary and force plots showed that non-responding patients had lower “CET1w(3D)_firstorderM” values than responding patients, likely due to the lack of a gadolinium-based contrast agent caused by poor vascular supply, helping to understand the radiomic model’s pathophysiological mechanism [30]. Laifa et al developed an MRI-based noninvasive radiomic model for preoperative glioma grading [69]. The results showed that radiomic features selected by SHAP reflected image heterogeneity, potentially indicating stronger proliferation ability, more cystic degeneration or necrosis, and higher hemorrhage frequency [69]. The aforementioned traditional radiomic studies are detailed in Supplementary Table 1.

## The interpretability of deep learning radiomics in brain tumor

### Concept learning models

**Fig. 5:**
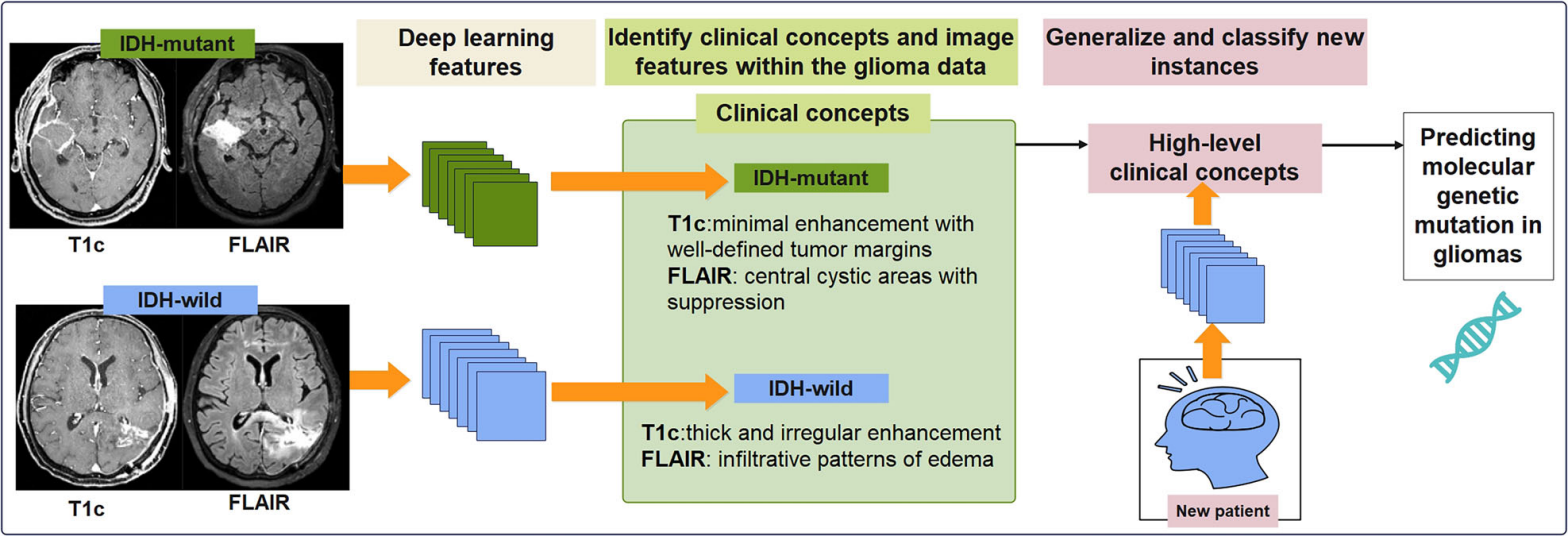
Concept learning model in brain tumor. Concept learning model focuses on understanding the underlying patterns and relationships in data, allowing the model to classify new instances based on learned features

Concept learning models sought to understand and categorize data based on given examples (high-level concepts) [70]. This method operates by training the radiomic model to recognize abstract, clinically relevant concepts in the imaging data. Chang et al developed a CNN to predict molecular genetic mutation status in gliomas, as shown in Fig. 5. Typically, IDH-mutant tumors showed absent or minimal enhancement with well-defined margins on T1c or central cystic areas with fluid attenuated inversion recovery (FLAIR) suppression. Conversely, IDH wild-type tumors exhibited thick and irregular enhancement or thin, irregular peripheral enhancement on T1c, and infiltrative edema patterns on FLAIR [71]. A clinician would observe that gliomas with irregular enhancement and infiltrative edema are more aggressive. Although clinicians can understand high-level clinical concepts, concept learning faces the issue of additional annotation costs, and the learned concepts may encode unintended information due to information leakage [70–72].

### Case-based reasoning

**Fig. 6:**
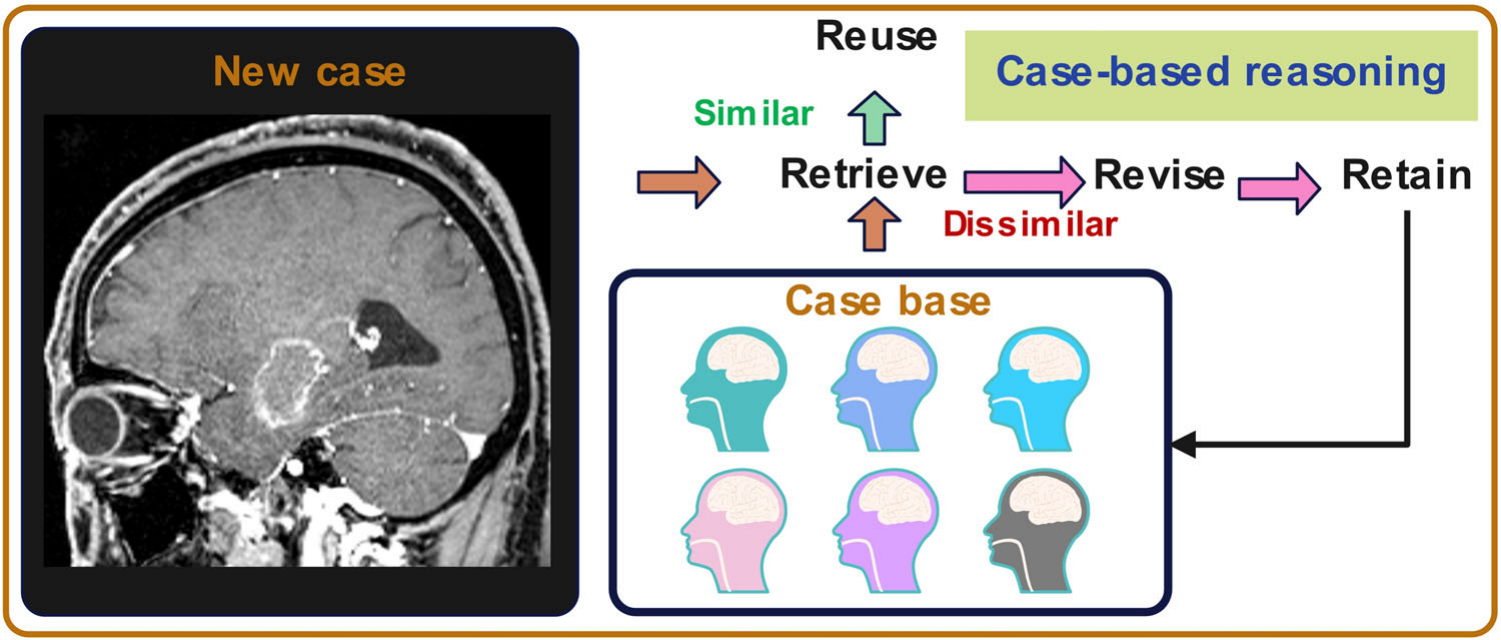
The process of case-based reasoning in brain tumor

Case-based reasoning (CBR) is a problem-solving approach that compares features from an input image against class-discriminative prototypes. Instead of generating solutions from scratch, CBR systems retrieve similar cases from a database of previously solved cases and adapt their solutions to fit the current problem [73]. The process of CBR includes building the case database, matching the new case, and generating a prediction and explanation through similar cases (as shown in Fig. 6). CBR in radiomics offers an intuitive, interpretable method that aligns with how clinicians often reason through cases—by referencing similar past experiences.

Sanja et al improved the CBR system for brain tumor radiotherapy treatment planning. They used neural networks and a naive Bayes classifier in the adaptation process to learn how differences in attribute values between retrieved and new cases affected the output [74]. The CBR system improved its success rate by 29% by adapting beam number and angles based on case base retrieval. However, it focused on beam number matching, while medical physicists evaluate treatment plans based on dose homogeneity. Future evaluations by medical physicists are recommended [74].

ProtoPNet uses prototypes, representative examples of features from the training data. These prototypes are used to make predictions by comparing new inputs to them, enhancing the interpretability of the predictive model [75], similar to CBR. Chong et al developed InterNRL, a framework consisting of a student-teacher model where the student is an interpretable prototype-based classifier (ProtoPNet) [76]. InterNRL achieved superior brain tumor segmentation results compared to other methods. Their findings showed that non-tumor prototypes captured healthy brain structures, while tumor prototypes focused on regions with abnormal brain tumors, aligning with clinical diagnostic criteria [76].

Although class-discriminative prototypes are learned and final classification involves comparing features from input images with the prototypes, CBR is difficult to train due to susceptibility to noise and compression artifacts.

### Counterfactual explanation

**Fig. 7:**
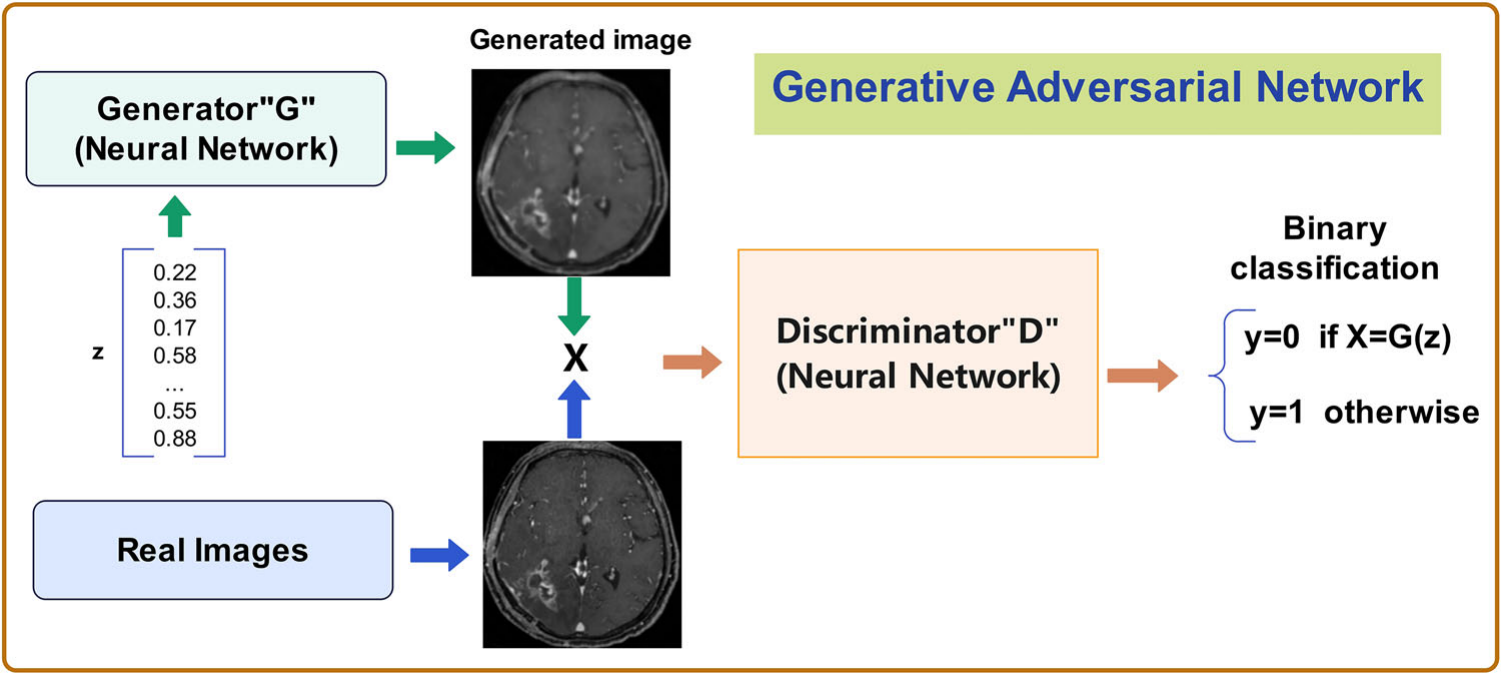
The process of generative adversarial networks in brain tumor radiomics

Counterfactual explanations (as shown in Table 3) show how slight changes in the input could lead to different outcomes, revealing the conditions under which the model’s decision changed [77]. Counterfactual explanations work by identifying the minimal changes needed to an input (e.g., tumor size, shape, or texture) to flip the model’s decision from one outcome to another (e.g., from benign to malignant). These explanations could use Generative Adversarial Networks (GANs), a type of DL model for generating synthetic data indistinguishable from real data. GANs consisted of two neural networks, as shown in Fig. 7, a generator and a discriminator, trained simultaneously through adversarial processes [78].

**Table 3:** The process of interpretability methods in radiomics

Interpretability methods	Counterfactual explanation	Latent space interpretation	Internal network representation	Language description
Overview	Shows what minimal changes to input features would alter the prediction.	Interprets the compressed feature space in which the model groups similar cases.	Examines how each network layer processes input data to reveal how the model recognizes important features.	Translates these complex, abstract features into human-readable language.
Step 1	**Initial Prediction**: The model predicts an outcome based on current radiomic features (e.g., size, shape, or texture).	**Feature Compression**: The model compresses input radiomic features (e.g., tumor texture, shape) into a smaller latent space.	**Layerwise Representation**: As radiomic features pass through each layer of the network, the model progressively extracts more abstract patterns.	**Feature Extraction**: The model first extracts radiomic features from medical images, such as a tumor’s size, shape, and texture.
Step 2	**Generating Counterfactuals**: The model identifies what small modifications to these features (e.g., reducing size, smoothing texture) would lead to a different prediction.	**Interpretation of Latent Space**: By interpreting clusters or patterns in the latent space, clinicians can understand how the model perceives relationships between different radiomic features and outcomes.	**Feature Importance by Layer**: Internal network representation techniques analyze the hidden layers to determine what features are being prioritized at different stages of the decision-making process.	**Mapping Features to Descriptive Language**: The model then translates these features into natural language by mapping specific radiomic patterns to well-understood clinical descriptions.
Step 3	**Minimal Changes**: The goal is to find the smallest changes that would switch the model’s prediction from one outcome to another.	**Mapping Back to Clinical Concepts**: The latent space is linked back to clinically relevant features to make it understandable for clinicians.	**Visualization**: These internal representations can be visualized to show what the model “sees” at each layer, helping clinicians understand how features like tumor texture or edge sharpness are interpreted.	**Providing a Clear Explanation**: After making a prediction, the model uses these language descriptions to explain why it reached that decision.

Rajeev et al used Cyclic-GAN to expand the brain tumor dataset for tumor detection and staging [79]. The Cyclic-GAN, consisting of two generators and two discriminators, to learn the pattern of brain tumor pixel mapping. This approach applied minimal perturbations to the original image to significantly change the classifier’s prediction, enhancing the dataset for image augmentation [79]. By applying transformers to GANs, predictive models demonstrated the ability to learn long-distance dependencies in global space. Liqun et al proposed a transformer-based GAN to automatically segment brain tumors using multimodal MRI. Their results showed that replacing CNN with transformer blocks in the bottleneck enhanced feature capture, improving pixelwise segmentation performance [80].

GANs have garnered significant attention for generating realistic data. However, the theoretical understanding of GANs remains limited, and the underlying biological and theoretical mechanisms behind their effectiveness are still an active area of research.

### Latent space interpretation

Latent spaces (as shown in Table 3), which are lower-dimensional representations, encode data to capture essential features and structures while reducing dimensionality. Latent variables, representing data in this space, contained compressed information [81]. The interpretation of latent space involved analyzing these internal representations, often using techniques like t-Distributed Stochastic Neighbor Embedding (t-SNE) to visualize high-dimensional data in two or three dimensions [82]. Yun et al employed t-SNE to visualize the distribution of radiomic features to mitigate tumor selection bias in characterizing brain tumor regions. The study found similar radiomic features distributions in the Wilcoxon signed rank test [83]. However, this method resulted in the loss of biological information when projecting the high-dimensional feature space to two dimensions.

Representation disentanglement in latent space involves separating distinct, interpretable factors of variation in data across different dimensions of latent space [81]. Recently, the effectiveness of anatomical and modality-specific information disentanglement in multimodal MRI cross-reconstruction was questioned regarding the true disentanglement of learned representations [84]. Given the high variability in brain morphology across individuals, Jiahong et al posited that anatomical representations from the same subject across different modalities should resemble more closely than those from the same modality but different subjects [85]. Consequently, they introduced a novel margin loss to regulate the similarity of within-subject cross-modality representations relative to across-subject within-modality similarity, addressing information leakage in existing disentanglement methods [85].

β-Variational Autoencoders (VAEs) introduce a hyperparameter β to balance the trade-off between reconstruction accuracy and disentanglement in the latent space [86]. Bilal et al proposed a framework that utilized VAEs and GANs to generate a substantial dataset of brain tumor images for training DL models, enhancing brain tumor classification results [87]. In their study, the VAE comprised an encoder-decoder network; the encoder converted an image into a latent vector, while the decoder used this vector to generate noise that encapsulated the brain tumor image manifold’s information [87]. Despite the potential for interpretability, latent spaces, especially in high-dimensional models, often remain complex and opaque, challenging direct interpretation of the encoded features.

### Internal network representation

Network Dissection is employed to interpret the internal representations of neural networks, particularly CNN [88]. It elucidates the concepts represented by individual neurons, enhancing the interpretability of the network’s operations and revealing the hierarchical structure of learned features from simple edges to complex object parts (as shown in Table 3) [88]. Parth et al explored various techniques to elucidate the functional organization of brain tumor segmentation models and to visualize internal concepts, clarifying how these networks achieve precise tumor segmentations [89]. The application of network dissection showed that filters within brain tumor segmentation networks could learn both explicit and implicit disentangled concepts such as whole tumor regions, edema regions, and the distinctions between white and gray matter [89].

### Language description

Symbolic emergent language (as shown in Table 3) refers to communication protocols that naturally developed in multi-agent systems [90], without pre-design or explicit programming. In these systems, agents developed efficient communication methods to optimize collective actions, improving interpretability [90]. Alberto et al applied this concept to brain tumor segmentation by introducing a generalized symbolic semantic framework involving two agents—a Sender and a Receiver [90]. The Sender network received input and generated a symbolic sentence, while the Receiver interpreted this sentence to produce a segmentation mask. This framework enabled the learning of clinically relevant language, distinguishing tissue types and object morphology [90]. However, emergent languages could pose interpretative challenges if the symbols were overly complex or abstract. The aforementioned DL radiomic studies are detailed in Supplementary Table 2.

### Attribution map

Gradient-weighted Class Activation Mapping (Grad-CAM) computes the gradients of the target class score relative to the feature maps of the last convolutional layer highlighting important regions in the input for specific predictions [91]. Francesco et al proposed a method for brain tumor detection and localization using MRI. Grad-CAM highlighted tumor regions in yellow and areas not relevant to pathology in purple [92]. Mingyang et al used Grad-CAM to visualize tumor segmentation with a pyramid structure, showing consistent identification of tumor core while focusing on different regions for detailed analysis [93].

Perturbation-based methods systematically perturb input data to interpret model predictions by observing the output effects. Unlike gradient-based approaches, these methods focused on changes in predictions as input features are modified. LIME approximates the model locally around a specific prediction with a simpler model by perturbing the input data and using changes in the prediction to fit a local surrogate model [66]. Rezuana et al developed an explainable deep neural network for brain tumor classification using MRI data and applied LIME to highlight significant image features that aligned well with clinically relevant model focus areas [94].

SHAP values, computed by considering all possible combinations of feature presence and absence, calculate the marginal contribution of each feature based on cooperative game theory [95]. Loveleen et al used a CNN with SHAP to predict brain tumor subtypes, showing how individual features impacted model output. Positive SHAP values (red pixels) increased class likelihood, while negative values (blue pixels) decreased it [96].

Trainable attention mechanisms, where neural networks dynamically focus on different parts of the input, allow models to weigh the importance of various input tokens for each output token. These mechanisms are particularly useful in tasks like image recognition and captioning, enabling focus on pertinent image sections [97]. David et al added attention mechanisms to a U-Net architecture for meningioma segmentation, improving the identification of global spatial relationships and areas unlikely to contain meningiomas [98].

Layerwise relevance propagation (LRP) is utilized to explain deep neural networks by decomposing a model’s prediction into contributions from each input feature, revealing which parts of the input most significantly impacted the model’s decision [99]. Hyungseob et al applied LRP to multiparametric MRI to generate clinical referral suggestions for brain lesions. The study revealed significant discrepancies between T1WI and DWI heatmaps, aiding in differentiating tumorous from non-tumorous conditions by highlighting non-overlapping areas in non-tumorous cases, enhancing interpretability [100].

Attribution maps visually illustrate the contribution of each input feature, increasing the transparency and trustworthiness of model predictions. However, these maps often offer local interpretations, which may not consistently apply across various inputs. The attribution map methods for interpreting DL radiomic studies in brain tumor are detailed in Supplementary Table 3.

## Recommendations on utilizing interpretability methods to pair with radiomic models

Interpretability techniques are integral throughout the radiomics modeling lifecycle- from model development to model validation, choosing suitable interpretability techniques is crucial. These techniques should be tailored to the specific challenges at hand, the intended audience (such as clinicians), and the current phase of model development. Accordingly, we present a structured guide in Table 4 to assist in identifying the most appropriate interpretability methods for different contexts.

**Table 4:** The structured guide in identifying the most appropriate interpretability methods for brain tumor radiomic studies

Interpretability methods	When to use	Why to use	Recommendation
Feature maps and activation maps	Model development stage	Ensure that the model is learning appropriate low- and high-level features.	Combine these maps with t-SNE or PCA to illustrate the relationships between the learned deep features and established biological or clinical categories.
Occlusion sensitivity or perturbation-based methods	Model development stage	Assess the impact of obscuring specific areas of the image on the model’s predictions and help determine whether the model is concentrating on clinically relevant regions.	Implement occlusion sensitivity in tasks such as tumor segmentation or the identification of critical features, such as necrotic or edematous areas in brain tumors.
Grad-CAM	Model refinement	Visualize the most significant areas of the image that influence the model’s classification or prediction.	It is especially useful in tasks that require high localization, such as pinpointing malignant areas.
Attention mechanisms	Model refinement	Models that need to assign varying importance to different regions of the image.	Incorporate attention layers when working with heterogeneous images, like tumors with diverse regions of interest, to enhance the model’s ability to learn clinically significant areas.
SHAP or LIME	Model validation	Provide a clear, quantitative explanation of which features contributed to each individual prediction	Employ SHAP in outcome-based assessments where it is vital to clarify the reasons behind the model’s assessment of elevated or reduced risk for particular patients. LIME can provide insights into individual predictions by approximating the behavior of the deep model with a simpler local model.
LRP	Model validation	Aim to trace through the network to assign relevance to specific areas of the input image for clinical decision-making	LRP offers pixelwise relevance, which is particularly beneficial in high-stakes medical diagnostics.
Correlation with handcrafted features	Model comparison (Deep vs. handcrafted features)	Verify that the features learned by the deep learning model align with clinically relevant handcrafted features, such as texture, intensity, or shape.	Employ correlation analysis to illustrate the relationship between deep features and established biomarkers or radiomic characteristics.
Dimensionality reduction	Model comparison	Visualize deep features in a reduced-dimensional space to assess how effectively the features differentiate between various patient groups or clinical outcomes.	Utilize t-SNE or PCA to examine how deep features cluster patients according to significant clinical outcomes.

## Discussion

According to explainable AI taxonomies, methods such as textual explanations can be used to explain ML or DL prediction models [52, 53]. However, few explanation methods have been applied to brain tumor radiomics, and many other interesting explainable methods are worth exploring.

For evaluating explainability methods in brain tumor radiomics, some literature and scoring standards provide specific criteria. Item #43 of the CheckList for EvaluAtion of Radiomics research evaluates interpretability and explainability methods, requiring radiomic studies to describe techniques used to enhance model interpretability and explainability [101]. The Radiomics Quality Score 2.0 includes items for explaining both handcrafted radiomics and DL-based models [4]. One approach is to detect and discuss biological correlates. In this study, we listed the physiopathologic mechanisms of the model to deepen the understanding of radiomics in brain tumors. Another aspect involves detailing intrinsic or post hoc interpretability methods or uncertainty estimation methods used. We also provided an overview of the methods used in brain tumor radiomics. However, few studies have evaluated these explanations using in-silico trials or clinician input. A common challenge in brain tumor radiomics is that most studies focus on the model and algorithmic level for interpretation. The explanation of biological mechanisms requires further validation through animal models or molecular pathology experiments. Therefore, more innovative experimental designs are needed to elucidate the biological interpretability of radiomics in the future.

In radiomics interpretability studies, a trade-off between model interpretability and performance was emphasized [102]. High-performance models, such as deep neural networks, usually have complex structures and numerous parameters. While these models captured intricate patterns and nonlinear relationships, their internal workings were difficult to interpret, resulting in lower interpretability. In contrast, simpler models, like linear regression, were easier to interpret due to their straightforward structures and fewer parameters but often had insufficient performance, especially with high-dimensional and complex data. Clinicians needed to balance interpretability and performance based on the application scenario and requirements. In some cases, model interpretability was more important than achieving the highest performance. Additionally, standardized and reproducible methods were needed in clinical practice to evaluate and enhance the interpretability of brain tumor radiomics [103]. To achieve clinical translation of brain tumor radiomics models, multidisciplinary collaboration among researchers, neurosurgeons, neuro-oncologists, radiologists, machine learning engineers, and the human-computer interface community was crucial. This collaboration was essential for designing brain tumor radiomics models that were both effective and interpretable.

## Conclusions

By analyzing vast amounts of imaging data, radiomics enables the extraction of quantitative features to aid in brain tumor diagnosis, prognosis, treatment planning, and other clinical management. This study incorporated biological domain knowledge and interpretability methods, allowing clinicians to understand and trust the insights from complex imaging data. Ultimately, this enhanced decision-making and patient care in neuro-oncology.

## Supplementary information

ELECTRONIC SUPPLEMENTARY MATERIAL

## Abbreviations

18F-DOPA-PET: 18F-DOPA positron emission tomography
AI: Artificial intelligence
AUC: Area under the curve
BM: Brain metastasis
CBR: Case-based reasoning
CNN: Convolutional neural network
DL: Deep learning
DWI: Diffusion-weighted imaging
EGFR: Epidermal growth factor receptor
FLAIR: Fluid attenuated inversion recovery
GANs: Generative adversarial networks
GBM: Glioblastoma
Grad-CAM: Gradient-weighted class activation mapping
LIME: Local interpretable model-agnostic explanations
LRP: Layerwise relevance propagation
MGMT: O6-methylguanineDNA methyltransferase
ML: Machine learning
MRI: Magnetic resonance imaging
NSCLC: Non-small cell lung cancer
OS: Overall survival
PFS: Progression-free survival
RIBI: Radiation-induced brain injury
RRS: Radiomic risk score
SHAP: Shapley additive explanations
SRS: Stereotactic radiosurgery
t-SNE: t-distributed stochastic neighbor embedding
VAEs: Variational autoencoders
WSIs: Whole-slide images
