# Supplementary material for: The clinical implications and interpretability of computational medical imaging (radiomics) in brain tumors
Source: Insights Imaging. 2025 Mar 30;16:77. doi: 10.1186/s13244-025-01950-6 (PMC11955438; doi:10.1186/s13244-025-01950-6)
Supplement: Supplementary file 1 — ELECTRONIC SUPPLEMENTARY MATERIAL [file 13244_2025_1950_MOESM1_ESM.pdf]

The clinical implications and interpretability of computational medical imaging (radiomics) in brain tumors

ELECTRONIC SUPPLEMENTARY MATERIAL

Insights Imaging (2025) Wang Y, Hu Z, Wang H.

Supplementary Table 1 The interpretability of traditional radiomic studies in brain tumor.

Reference	Image modality	Study content	Machine learning methods	Interpretation methods	Domain knowledge	Physiopathologic mechanism of radiomic model
[59]	FLAIR	Predict the response to bevacizumab in patients with brain necrosis after radiotherapy.	Multivariable logistic regression	Transparent model (Intrinsic; Specific; Global)	The radiomics signature consisted of 18 selected features and showed good discrimination performance. Response score= 3.436 × radiomics score - 0.024× IRB- 0.043×IBT + 0.451.	Cannot provide a biological semantic analysis of radiomic features for predicting the response.
[60]	contrast-enhanced MRI	Differentiate brain pilocytic astrocytoma from glioblastoma.	Decision tree model	Transparent model (Intrinsic; Specific; Global)	Entropy and SumAverg were included in the model.	These selected co-occurrence matrix features have some relationship with the enhancement difference of brain pilocytic astrocytoma and glioblastoma.
[62]	Gadolinium-T1WI, T2WI, FLAIR	Differentiate poor and improved PFS in glioblastoma.	A linear discriminant analysis classifier	Transparent model (Intrinsic; Specific; Global)	Radiomic features from the T2/FLAIR hyperintensity subcompartment were the most stable and discriminatory of PFS in GBM tumors compared with the features from enhancing tumor and necrotic core.	Cannot illustrate the physiopathologic mechanism of radiomic model for differentiate the PFS.
[63]	enhanced T1WI, T2WI, FLAIR	Predict the progression-free survival (PFS) in glioblastoma.	A linear combination and multiplied with their respective coefficients to construct a RRS, Cox proportional hazard regression model for predicting PFS	Habitat analysis	RRSs used radiomic features from the tumor habitat were different across low-risk and high-risk groups.	Laws features from enhancing tumor were associated with the extent of infiltrating tumor and hyperplastic blood vessels in cellular tumor. CoLIAGe inverse different moment feature from the enhancing tumor was found to be significantly correlated with the perinecrotic zone in cellular tumor.
[64]	pretreatment Gadolinium-T1WI, T2WI, FLAIR	Create a survival risk score to predict PFS in glioblastoma and obtain a biological basis for these prognostic radiomic features.	Radiomic features picked by the LASSO and then pooled in a linear combination and multiplied with their respective coefficients to construct an RRS, Cox regression model for predicting PFS	Habitat analysis	RRS used 25 Gd-T1w radiomic features resulted in statistically significant KM curves.	Significant correlations were found between the shape features of the peritumoral edema region and biological processes of cell proliferation, angiogenesis, and cell adhesion. Laws energy and Gabor wavelet texture features from within the peritumoral edema region were correlated with cell proliferation and angiogenesis.
[67]	Whole Slide images	Provide an automated glioma grading platform.	RF, SVM, GBDT, NN	LIME (Post-hoc; Agnostic; Local)	The platform achieved the highest grading accuracies of 0.91 ± 0.08, 0.90 ± 0.08, and 0.90 ± 0.07 for grade II, III, and IV gliomas, respectively.	LIME provided an explanation for each individual grading result. Morphological features including the standard deviation of cells' max axis and perimeter were also found to be a significant contributor to glioma grading because HGG often exhibits strong heterogeneity with irregular cell shapes.
[68]	DSC–MRI	Predict IDH mutations in gliomas.	SVM, RF, KNN, a logistic regression, AdaBoost	LIME, SHAP (Post-hoc; Agnostic; Local)	DSC–MRI radiogenomics provided increased predictive performance from models and provided understandable patterns between IDH mutation status and the extracted features.	The potential intuitive correlations between the IDH–wildtype increased heterogeneity and the texture complexity.
[30]	CET1-w 3D	Assess the treatment response of WBRT.	SVM	SHAP (Post-hoc; Agnostic; Local)	The radiomic-clinical model with the SHAP method can be useful for assessing the treatment response of WBRT.	Patients in the non-responding group had low "CET1-w(3D)_firstoderM" than patients in the responding group, this may due to the lack of a gadolinium-based contrast agent due to poor vascular supply.

[69]	CE-T1WI	Preoperative gliomas grading.	XGBoost	SHAP (Post-hoc; Agnostic; Local)	By integrating SHAP values, the model uncovered an interaction pattern between radiomics features and pathological grade.	First-order and GLCM features extracted from the image after LoG and wavelet filtering are the most important factors for identifying gliomas. And these features identified can indeed potentially reflect stronger proliferation ability, more cystic degeneration or necrosis, and higher frequency of hemorrhage.

Supplementary Table 2 The interpretability of deep learning radiomic studies in brain tumor.

Reference	Image modality	Study content	Machine learning methods	Interpretation methods	Domain knowledge	Physiopathologic mechanism of the model
[71]	FLAIR, CE-T1WI	Predict molecular genetic mutation status in gliomas.	CNN	Concept learning models (Intrinsic；Specific; Local)	Each genetic category was associated with distinctive imaging features such as definition of tumor margins, T1 and FLAIR suppression, extent of edema, extent of necrosis, and textural features.	IDH-mutant tumors showed minimal enhancement with well-defined tumor margins on CE-T1WI, or central cystic areas with FLAIR suppression. Conversely, IDH wild-type tumors exhibit thick and irregular enhancement or thin, irregular peripheral enhancement on CE-T1WI, and infiltrative patterns of edema on FLAIR.
[74]	CT	Improve the performance of brain tumor radiotherapy treatment planning.	Neural networks and naive Bayes classifier	Case-based reasoning (Intrinsic；Specific; Local)	The CBR system compared the beam number and beam angles suggested by the retrieval/adaptation process with the beam number and beam angles initially suggested by the medical physicists and stored in the cases base. And the adaptation-guided retrieval approach for beam number improved the success rate of the CBR system by 29%.	The model considered the retrieval to be successful when the retrieved cases have the same beam number or differ within a predefined threshold as suggested by the medical physicists. However, medical physicists evaluate the treatment plan based on the homogeneity of dose administered to cancer cells and the dose administered to non-tumor tissue.
[76]	BraTS 2019	Brain tumor segmentation.	InterNRL consisted of a prototype-based classifier (ProtoPNet) and an accurate global image classifier (GlobalNet).	Case-based reasoning (Intrinsic；Specific; Local)	InterNRL outperformed the existing best method, suggesting that the interpretable prototypes are also effective in the weakly-supervised segmentation task.	The non-tumor prototypes tended to capture healthy brain structures, while the tumor prototypes typically focused on regions with abnormal brain tumors. These findings aligned with the diagnostic criteria used by clinicians, indicating that the learned prototypes were both representative and discriminative.
[79]	Brain MRI	Brain tumor detection and classification.	InceptionResNetV2; RF; Cyclic-GAN was used to increase the dataset size.	Counterfactual explanation (Intrinsic；Specific; Local/Global)	Data Augmentation was employing through the Cyclic GANs to overcome data related problems which generated images that are comparable to the input data.	Cyclic-GAN to learn the pattern of brain tumor pixel mapping in datasets.
[80]	BRATS2015 dataset	Propose a novel transformer-based GAN for brain tumor segmentation.	The framework consists of a generator and discriminator for competing training	A transformer-based GAN (Intrinsic；Specific; Local/Global)	The generator exploited transformer with Resnet module in 3D CNN for segmenting multi-modalities MRI brain tumors.	For pixelwise brain tumor segmentation task, replacing CNN with transformer blocks on the bottleneck contributes to capturing more features from encoder.
[83]	Brain MRI	Characterize brain tumor regions.	RF, SVM, and back propagation neural network	t-SNE （Intrinsi；Specific; Local/Global）	t-SNE was used to visualize the data distribution so as to avoid tumor selection bias.	Loss of biological information.
[85]	Brain MRI; BraTS 2020 dataset	Brain MRI cross-reconstruction.	U-Net type model	Representation disentanglement （Intrinsic；Specific; Local/Global）	The proposed similarity regularization can decouple the modality-specific appearance features from the structural information shared between modalities.	As the brain’s morphological shape exhibits significant heterogeneity across individuals, anatomical representations derived from different imaging modalities of the same subject should display more similarity than those obtained from the same modality across different subjects.
[87]	Brain tumor MRI	Brain tumor classification.	ResNet50	VAEs and GAN （Intrinsic；Specific; Local/Global）	The VAEs method helps the GAN to avoid mode collapse and generate realistic-looking brain tumor magnetic resonance images.	The VAE consisted of an encoder-decoder network. The encoder took an image as input and generated a latent vector, while the decoder took this latent vector as input and produced noise. This noise was not random but carried information about the image manifold.

[89]	BraTS 2018 dataset	Brain tumor segmentation.	2D and 3D Deep Neural Networks	Network Dissection（Intrinsic；Specific; Local）	Brain tumor segmentation networks learned certain human-understandable disentangled concepts on a filter level and took a top-down or hierarchical approach to localizing the different parts of the tumor.	Individual filters of brain-tumor segmentation networks learned explicit as well as implicit disentangled concepts, such as, the concept whole tumor region, edema region, and the white and gray matter region.
[90]	TCGA dataset	Brain tumor segmentation.	A UNet-like architecture combined with a Sender and a Receiver	Symbolic emergent language（Intrinsic；Specific; Local/Global）	The framework suggested direct interpretation of the symbolic sentences to discriminate between normal and tumor tissue, tumor morphology, and other image characteristics.	The results suggested the feasibility of associating symbolic sentences with clinically relevant information, such as tissue type (tumor vs. normal), object morphology (area, eccentricity), object localization (tumor laterality and location), and tumor histology and genomics data.

Supplementary Table 3 The attribution map methods for interpreting deep learning radiomic studies in brain tumor.

Reference	Image modality	Study content	Machine learning methods	Interpretation methods	Domain knowledge	Physiopathologic mechanism of the model
[93]	Brain Tumor Detection 2020	Brain cancer detection and localization.	VGG16, ResNet50, Alex_Net, MobileNet	Grad-CAM (Post-hoc; Specific; Local)	By applying Grad-CAM, the features or patterns that the model considers indicative of a brain tumor or pathology were provided.	Grad-CAM assigned a yellow color to help identify regions in the image that were particularly symptomatic of a brain tumor or indicative of pathology. Conversely, Grad-CAM used a purple color to indicate areas that were not of interest for the detection of pathology.
[94]	BraTS2018	Brain tumor segmentation.	ResNet	Grad-CAM (Post-hoc; Specific; Local)	The pyramid structure could extract features of different scales from the input image by pooling the extracted features in multiple dimensions and then concatenate these features to obtain the final multiscale features.	Visualization results at different scales demonstrated consistency in easily identifying tumor core parts while focusing on varying regions for other details.
[95]	Brain Tumor MRI Dataset	Brain tumors classification.	VGG19 incorporates the Inverted Pyramid Pooling Module	LIME (Post-hoc; Agnostic; Local)	LIME highlighted the features or areas focused on while predicting individual images.	LIME displayed significant features of the images by highlighting regions with a high degree of alignment between the model's focus and clinically relevant features.
[97]	Brain MRI	Predict discrete subtypes of brain tumors.	CNN	SHAP (Post-hoc; Agnostic; Local)	The effect of individual input features on the model's output was clearly explained using SHAP.	Positive SHAP values, which increased the likelihood of the class, were represented by red pixels. In contrast, negative SHAP values, which decreased the probability of the class, were represented by blue pixels.
[99]	CE-T1WI	Meningioma segmentation.	U-Net	Trainable attention (Intrinsic; Specific; Local)	The effect of attention schemes had been observed whereby predictions appeared to be restricted to the brain itself or its close boundaries.	The model learned global spatial relationships to define certain no-prediction zones where meningiomas were unlikely to occur.
[101]	CE-T1WI, FLAIR, DWI	Discriminate for intra-axial mass-like brain lesions.	U-Net	LRP (Post-hoc; Specific; Local)	Solid portions of tumors showed a high overlap of relevance.	Discordance between CE-T1WI and DWI heatmaps proved most helpful for differentiating non-tumorous conditions from tumorous ones, as high-relevance areas on CE-T1WI and DWI did not align in non-tumorous conditions.
